# Pharmacoeconomic burden in the treatment of psoriatic arthritis: from systematic reviews to real clinical practice studies

**DOI:** 10.1186/1471-2474-15-25

**Published:** 2014-01-20

**Authors:** Ennio Lubrano, Antonio Spadaro

**Affiliations:** 1 Academic Rheumatology Unit, Department of Medicine and Health Sciences, University of Molise, Campobasso, Italy; 2 Dipartimento di Medicina Interna e Specialità Mediche - UOC di Reumatologia, “Sapienza” - Università di Roma, Azienda Policlinico Umberto I, Viale del Policlinico 155, 00161 Rome, Italy

## Abstract

The economic assessment of treatment options in a chronic and severe disease like Psoriatic Arthritis (PsA) is crucial to estimate the burden of costs. In particular, the impact of new costly medications such as biologic agents have been studied to figure this important aspect of a multifaceted disease. In a previous observational, longitudinal multicentre cost evaluation study, the results showed that biologic agents are cost-effective. This study was obtained from the real clinical practice and encompassed PsA patients refractory to traditional treatments. Similar data were also obtained from reviews analysis of Randomized Controlled Trials (RCTs). Recently, Cawson et al. performed a systematic review, network meta-analysis and economic evaluation of biological therapy for the management of active PsA. The review was conducted to identify relevant, recently published studies and the new trial data were synthesized, via a Bayesian network meta-analysis (NMA), to estimate the relative efficacy of the TNF-α inhibitors in terms of Psoriatic Arthritis Response Criteria (PsARC) response, Health Assessment Questionnaire (HAQ) scores and Psoriasis Area and Severity Index (PASI). In particular the analysis showed that, on average, etanercept was the most cost-effective treatment and, at the National Institute for Health and Care Excellence willingness-to-pay threshold of between £20,000 to £30,000, etanercept is the preferred option. This study, as a systematic review, has been focused on main RCTs on active PsA treated by biological DMARDs and limitations to this analysis arise from a paucity of data on long-term follow up, as well as radiological progression and long-term safety. These interesting results reflected the important role of biologic agents in the management of PsA, highlighting their efficacy and cost-effectiveness. However, there are some unmet needs for pharmacoeconomic considerations based on prospective and/or on real clinical practice studies, as well as considering all the intriguing aspects of this challenging disease.

## Commentary

Psoriatic Arthritis (PsA) is a chronic inflammatory disease characterized by musculoskeletal and skin manifestations, and variably associated with other extra-articular manifestations, showing a combination of destructive changes (joint erosions, tuft resorption, osteolysis) with bone proliferation (including periarticular and shaft periostitis, ankylosis, spur formation and non-marginal syndesmophytes) [1]. PsA has to be considered a potentially disabling disease which requires aggressive and continuous treatment; in a prospective study on early PsA, 47% of the patients showed the development of erosive changes within 2 years of diagnosis [2].

In the context of this complex disease, there is some evidence showing that peripheral joint involvement is progressive in the majority of PsA patients [3]. PsA has showed to be marked by increased disability [4], comorbidities [5] and high direct and indirect costs [6]. While treatment strategies have improved globally some clinical manifestations in recent years, there is still a lack of consensus regarding the role of traditional Disease-Modifying Anti-Rheumatic Drugs (DMARDs) in controlling the progression of structural damage, as well as both in the long-term disease control [7]. The introduction of new biological molecules, such as etanercept, for the treatment of PsA has modified the management of this disease reaching a good clinical control of the disease [8-10]. Nevertheless the role of combination therapy in PsA has been not defined in term of improvement of effectiveness and safety compared to biologic monotherapy, even if etanercept showed promising results when associated to methotrexate [11] or cyclosporine [12]. Another conflicting issue is the role of biologic agent in treating predominant axial subset of PsA, and, for instance, etanercept showed to be effective in the axial subset of the disease [13].

However, biologic agents are costly medications, not easily available to all patients with some restriction from the various Health Systems and private insurances. Moreover, some PsA patients may experience adverse effects, and not all patients respond adequately requiring sometimes the switch to another biologic agent [14].

All these aspects paved the way to pharmacoeconomic considerations [15] and in the last few years some studied have been carried out to estimate the burden of costs of biologic agents. In 2008, an observational, longitudinal multicentre cost evaluation study was carried out looking at PsA patients refractory to traditional treatment [16]. The results showed that biologic agents are cost-effective. This study was obtained from the real clinical practice and encompassed PsA patients refractory to traditional treatments, assessing retrospectively for 6 months previously the onset of biologic agents and prospectively for other 6 months [16].

In a recent issue of the journal [17], Cawson et al. presented the data from a new economic evaluation supported by an updated systematic review and meta-analysis that included recent data for all four TNF-α inhibitors (infliximab, etanercept, adalimumab and golimumab) with the aim to determine the relative cost-effectiveness of all UK licensed for the treatment of active, progressive PsA in patients with inadequate response to previous DMARDs [17]. The meta-analysis results were used in a revised economic model which updates the previous NICE models [18,19] to provide a cost-effectiveness comparison of all four TNF-α inhibitors. The authors concluded that biologic agents were cost-effective for treating patients with active PsA compared to traditional treatments. In particular, etanercept is cost-effective compared to the other biologic treatments [17].

These findings reported by Cawson et al. confirm the crucial role of all biologic agents in controlling the clinical manifestations of the disease, showing an overall cost-effectiveness of these medications. Indeed, there are some limitations from this evaluation related to the type of analysis based only on RCTs without assessing the cost-effectiveness in real practice settings. Moreover, the wide spectrum of the PsA could be a potential bias for the cost-effectiveness studies. In fact, the heterogeneous clinical spectrum of the disease with a potential predominant pattern of the skin, nail and articular involvements should be taken into account for the pharmaco-economic evaluations, since the difficulties to assess these components by the actual outcome measures [20-22]. In particular the assessment of nail involvement [20] and radiographic evaluation of axial features of PsA [21,22] represent relevant tools in achieving the correct impact of pharmaeconomic burden of the disease. In fact, the axial involvement of PsA still represents a challenge in the management of this intriguing disease and, potentially, biologic agents are the most effective agents in treating this subset [23].

Another potential bias of this economic evaluation is the short term analysis considered and based on RCTs. Condition such as PsA, chronic by definition and with a clinical course most of the time characterized by different clinical findings, needs to be carefully evaluated in a long term period of time. At the present a five-year follow up study have been proposed as preliminary data, based on the real clinical practice (data not shown). In particular, this study, showing some preliminary data on a small group of PsA patients encompassing all the wide spectrum of the disease should be followed by larger multi-centre studies obtained from the daily outpatients setting to evaluate all the burden involved in the management.

Finally, pharmaeconomic analysis of combination (DMARDs plus TNF alpha blockers) treatment should be taken into account for a comprehensive estimation of cost-efffectiveness.

## Conclusion

At present, studies on cost-effectiveness of biologic agents in PsA have shown that these medications offer a good value for money. However, since there are no head-to-head studies on these biologic medications published yet, all the pharmaco-economic evaluations have been extrapolated from RCTs and just a few longitudinal studies based on real clinical practice. All the results obtained from these analysis have considered most of the intriguing aspects of PsA, demonstrating that biologic agents were effective on either skin and articular components of the disease, and cost-effective.

There is still an unmet need in obtaining pharmaco-economic analysis from a large population of PsA patients observed for long period of time in real clinical practice settings.

## Pre-publication history

The pre-publication history for this paper can be accessed here:

http://www.biomedcentral.com/1471-2474/15/25/prepub
